# Cost-effectiveness analysis of elacestrant versus standard endocrine therapy for second-/third-line treatment of patients with HR+/HER2- advanced or metastatic breast cancer: a US payer perspective

**DOI:** 10.3389/fonc.2023.1272586

**Published:** 2023-12-19

**Authors:** Wanxian Zeng, Xueqiong Cao, Jingwen Lin, Bin Zheng, Na Li, Maobai Liu, Hongfu Cai

**Affiliations:** ^1^Affiliated Union Hospital of Fujian Medical University, Fuzhou, Fujian, China; ^2^The School of Pharmacy, Fujian Medical University, Fuzhou, Fujian, China

**Keywords:** cost-effectiveness, elacestrant, partitioned survival model, advanced breast cancer, oncology

## Abstract

**Background:**

This study evaluated the cost-effectiveness of elacestrant (ELA) and standard-of-care (SOC) as second-/third-line treatment for pretreated estrogen receptor (ER)– positive/human epidermal growth factor receptor 2 (HER2)–negative advanced or metastatic breast cancer (A/MBC) in the US.

**Methods:**

The 3 health states partitioned survival model (PSM) was conducted from the perspective of the US third-party payers. The time horizon for the model lasted 10 years. Effectiveness and safety data were derived from the EMERALD trial (NCT03778931). Costs were derived from the pricing files of Medicare and Medicaid Services, and utility values were derived from published studies. One-way sensitivity analysis as well as probabilistic sensitivity analysis were performed to observe model stability.

**Result:**

ELA led to an incremental cost-effectiveness ratio (ICER) of $8,672,360/quality-adjusted life year (QALY) gained compared with SOC in the overall population and $2,900,560/QALY gained compared with fulvestrant (FUL) in the ESR1(estrogen receptor 1) mutation subgroup. The two ICERs of ELA were significantly higher than the willingness-to-pay (WTP) threshold values of $150,000/QALY.

**Conclusions:**

ELA was not cost-effective for the second-/third-line treatment of patients with ER+/HER2–A/MBC compared with SOC in the US.

## 1 Introduction

Breast cancer (BC) is one of the most commonly diagnosed cancers (11.7% of total cases) and the leading cause of cancer-related death among women globally (1). Since 2020, BC represents the second most diagnosed cancer (2), becoming the leading cause of cancer death among women aged 20–49 years in this year (3). According to the National Cancer Institute, BC is the most common cancer in US women except for nonmelanoma of the skin, accounting for 15% of new annual female cancer cases today (2). In addition, it is the second leading cause of cancer death among women in the US. Since 2004, The incidence rates of invasive breast cancer continue to increase by about 0.5% per year. As of January 1, 2022, there were approximately 4.1 million women with a history of breast cancer living in the United States, and approximately 4% of them present with metastatic disease (4). The survival of breast cancer patients differs from the stage at the time of diagnosis (4). A/MBC remains a virtually incurable disease, with a median overall survival (OS) of about 3 years and a 5-year survival rate of around 25%, even in countries without major accessibility problems (5).

After diagnosing BC, the neoplasm will be further checked for the expression of biological markers, which jointly define the subtypes of BC (2). Such as ER, progesterone receptor (PR), and HER2 (5). ER-positive/HER2-negative are the most common subset of breast cancers, accounting for 65% of cases of breast cancer among women less than 50 years of age and 75% of cases among older women (6).

29% of A/MBC women were originally diagnosed with IV-stage cancers (4). 60% of patients with stage IV BC receive noncurative-intent radiation and/or chemotherapy, but the efficacy is limited, and the prognosis is poor. A recent clinical study by Khan SA et al. (7) found that the survival rate of women with metastatic disease did not benefit from surgery of the primary tumor. Whereas, further expansion to targeted therapies, especially for HR-positive and HER2-positive disease, has improved survival for the metastatic disease over the past 3 decades (6, 8, 9). So far, the National Comprehensive Cancer Network Clinical Practice Guidelines in Oncology (NCCN Guidelines) (10) recommend endocrine therapy, with either aromatase inhibitors (AIs) or FUL, plus a cyclin-dependent kinase 4/6 (CDK4/6) inhibitor as first-line SOC for locally metastatic ER–positive/HER2–negative breast cancer, and sequential endocrine therapy or tamoxifen as a way of later-line therapy. However, endocrine monotherapy had shown limited activity in patients who have received prior CDK4/6 or mammalian targets of rapamycin inhibition (11). Novel therapeutic strategies that target this condition must be developed to address an important unmet clinical need for the vast majority of patients currently on A/MBC therapy.

ELA was a novel, oral selective ER degrader that demonstrated activity in early studies (12–14). What’s more, ELA was the first oral SERD that has demonstrated improved efficacy compared to SOC endocrine therapy in patients with advanced breast cancer. In 2002, FUL was approved for patients with ER-positive metastatic breast cancer. It has been almost 20 years since this last type of endocrine therapy was approved. On January 27, 2023, ELA was approved by the Food and Drug Administration (FDA) to treat postmenopausal women and adult men living with A/MBC that has tested positive for an ESR1 mutation with disease progression following treatment with at least one hormonal therapy based on the EMERALD clinical trial (15). Mutations in ESR1 gene lead to estrogen-independent ER activation. As a result, resistance to AIs but not ER inhibitors (e.g. selective ER degraders [SERDs] and selective ER modulators). The subgroup was included to compare the effectiveness of treatment between different groups of patients with detectable ESR1 mutations (16). EMERALD was an international, randomized, open-label, active-controlled, Phase III clinical study (11) (NCT03778931) accessing the efficacy and safety of an investigational oral hormone therapy, ELA (RAD1901), to the SOC hormone therapy options of FUL or an AI in patients with A/MBC that expresses the ER-positive and does not express HER2. In the EMERALD trial, patients treated with ELA had better progression-free survival (PFS) than patients treated with FUL. In addition to improved efficacy, ELA provides an oral treatment option instead of FUL’s intramuscular injection. The results showed that patients receiving ELA had superior PFS compared with those receiving SOC (in overall population = 2.8 months *vs.* 1.9 months or ESR1 mutation cohort = 3.8 months *vs*. 1.9 months). However, both groups experienced an initial decrease in PFS, the single median PFS in the overall population or ESR1 mutation cohort may not be sufficient to measure efficacy. Rather, more importantly, hazard ratio (HR) and landmark analyses at 6 and 12 months were used to assess efficacy over a longer period in this population. The HR reflected a 30% reduction in progression or death in the entire cohort and a relative reduction of 45% in the ESR1-mutant cohort. Landmark analyses at 6 and 12 months showed that the use of ELA significantly improved PFS at these later time points. This exciting result may mark the beginning of a paradigm shift in oral SERD therapy for estrogen receptor-positive breast cancer (17). ESR1 mutations result in estrogen-independent endoplasmic reticulum activation and therefore resistance to AIs, but not to endoplasmic reticulum inhibitors (e.g., selective endoplasmic SERDs and selective endoplasmic reticulum modulators). And in patients who have previously received CDK4/6 or mammalian target of rapamycin inhibition, ELA can fulfill this need for limited clinical activity of endocrine monotherapy (16). Although ELA has markedly contributed to A/MBC therapy, the high cost ($22511.06 for 30 tablets, 345mg per tablet) may be a heavy burden for patients and families. Thus, a cost-effectiveness analysis of ELA *vs.* SOC is necessary. The present study investigated the economic outcomes of implementing ELA or SOC regimens as a later-line therapy for patients who were previously treated, with estrogen receptor–positive/human epidermal growth factor receptor 2–negative advanced breast cancer from third-party payers in the United States. We provided the following articles according to the request of the CHEERS 2022 report list (18).

## 2 Methods

### 2.1 Cohort patients

The eligible population in this study utilized the sample characteristics of the EMERALD clinical trial: Participants were advanced/metastatic ER+/HER2- breast cancer; Their disease has progressed or relapsed on or after 1 or 2 lines of endocrine therapy, 1 of which was given in combination with a CDK4/6 inhibitor, for advanced or metastatic breast cancer; The ECOG PS 0 or 1 (11).

### 2.2 Interventions

According to the EMERALD clinical trial, the intervention group receives ELA 400 mg orally once daily, with reductions to 300 mg or 200 mg daily permitted for toxicity. The control group received SOC treatment, with FUL, anastrozole, letrozole, or exemestane monotherapy by per investigator’s choice. FUL was administered intramuscularly (IM) into the buttocks as 500mg dissolved into two 5 mL injections on C1D1 (cycle 1, day 1), C1D15, and C2D1 and Day 1 of every subsequent 28-day cycle; Anastrozole was given 1 mg/day orally on a continuous dosing schedule; Letrozole was given 2.5 mg/day orally on a continuous dosing schedule and exemestane was given 25 mg/day orally on a continuous dosing schedule, respectively (11). Since the clinical trial articles did not provide specific information regarding the percentage of each drug considered standard of care (SOC), except for mentioning that FUL was used in 165 patients (69.33%), we adopted the approach of assuming that the remaining three drugs in SOC were utilized equally (i.e., 30.67% * 1/3 = 10.22% of each drug).Treatment was continued until the disease progressed, the unacceptable adverse event, the withdrawal of consent, or the investigator’s decision, etc. Follow-up treatment was selected for patients who have progression of the disease, which was composed of anthracyclines, taxanes, anti-metabolites, vinca alkaloids, hormones, HER2-targeted therapies, and non-HER2-targeted therapies. The proportions of these therapies were derived from the study of Sorensen et al. (19, 20).

### 2.3 Model

#### 2.3.1 Model approach

The model of cost-effectiveness analysis (CEA) was based on a PSM that has three mutually exclusive health states (progression-free, post-progression, and death). The PSM uses the area under curves to represent the number of patients in each state. It is mainly used to evaluate the impact of interventions that can prolong the patients’ lives on their expected lifetime and quality of lives of the patients (21). Survival data in each arm were extracted in digital forms from the survival curves of EMERALD via GetData Graph Digitizer software (version 2.26;http://www.getdata-graph-digitizer.com/download.php). According to the method developed by Guyot et al. (22), Kaplan–Meier survival curves were reconstructed by R software (version 3.5.1) to obtain the new survival curves. There are 5 fitted distribution functions: Weibull, log-logistic, log-normal, Gompertz, and Gamma (23). Akaike information criterion (AIC), Bayesian information criterion (BIC), and visual simulation methods were used to check the goodness of fit. Thus, distribution functions with lower AIC and BIC and better visual simulation were selected as fitting curves, which were extrapolated to obtain long-term clinical survival results (24). The AIC and BIC values of the fitting results of each function were shown in **Supplementary Figures S1**, **S2**, and the selected fitting curves and data are shown in **Table 1**. The median PFS was in good agreement with the results observed in EMERALD (ELA PFS/FUL PFS: 3.37/1.94 *vs.* 2.8/1.9; 3.76/1.83 *vs.* 3.8/1.9), which ensure the practicability of the model (**Supplementary Figures S3, 4**).

**Table 1:** Model parameters and ranges used in the sensitivity analysis.

Variable	Baseline Value	Range		Reference
PFS survival model for all patients				
ELA (log-Normal)	meanlog=1.414933; sdlog=0.958343			
SOC (log-Logistic)	shape =2.24661; scale =2.73599			
FUL (log-Logistic)	shape =2.22577; scale =2.71317			
OS survival model for all patients				
ELA (log-Normal)	meanlog=3.192779; sdlog=0.851157			
SOC (log-Normal)	meanlog=3.09129; sdlog=1.09301			
PFS survival model for patients with ESR1 Mutation				
ELA (log-Normal)	meanlog=1.63382; sdlog=1.03730			
SOC (log-Logistic)	shape =2.42158; scale =2.57804			
FUL (log-Logistic)	shape =2.40911; scale =2.63315			
OS survival model for patients with ESR1 Mutation				
ELA (log-Normal)	meanlog=3.339774; sdlog=0.858537			
SOC (log-Normal)	meanlog=2.982833; sdlog=0.867711			
Drug cost, US $				
ELA per mg	2.175	1.088	2.610	(25)
FUL per 25mg	3.915	4.698	3.132	(26)
Anastrozole per mg	0.107	0.086	0.128	(27)
Exemestane per 25mg	0.713	0.571	0.855	(27)
Letrozole per 2.5mg	0.106	0.085	0.127	(27)
After progression	6,549	5,240	7,859	(20)
Subsequent treatment	9,061	7,248	10,873	(20)
End-of-life care	2,601	2,081	3,121	(20)
Follow-up visit	2,959	2,367	3,551	(28)
Administration	702	561	842	(29)
MAEs cost per event, First cycle only, US $				
Nausea	2,586	2,069	3,103	(30)
Back pain	2,501	2001	3001	(31)
Risk of MAEs in ELA (grade 3/4)				
Nausea	0.025	0.020	0.030	(11)
Back pain	0.025	0.020	0.030	(11)
Risk of MAEs in SOC (grade 3/4)				
Nausea	0.0090	0.0072	0.1080	(11)
Back pain	0.0040	0.0032	0.0048	(11)
Risk of MAEs in FUL (grade 3/4)				
Back pain	0.0060	0.0048	0.0072	(11)
QoL utility (per year)				
PF	0.837	0.753	0.921	(32)
PD	0.443	0.399	0.487	(33)
Disutilities of MAEs (per year)				
Nausea	0.05	0.02	0.10	(30)
Back pain	0.07	0.05	0.09	(34)
Other Parameters				
Discount rate	3%	0%	5%	(32)

MAEs, main adverse events; SOC, standard-of-care; OS, overall survival; ELA, elacestrant; FUL, fulvestrant; PD, progressed disease; PF, progression-free disease; PFS, progression-free survival.

#### 2.3.2 Model structure

**Figure 1:**
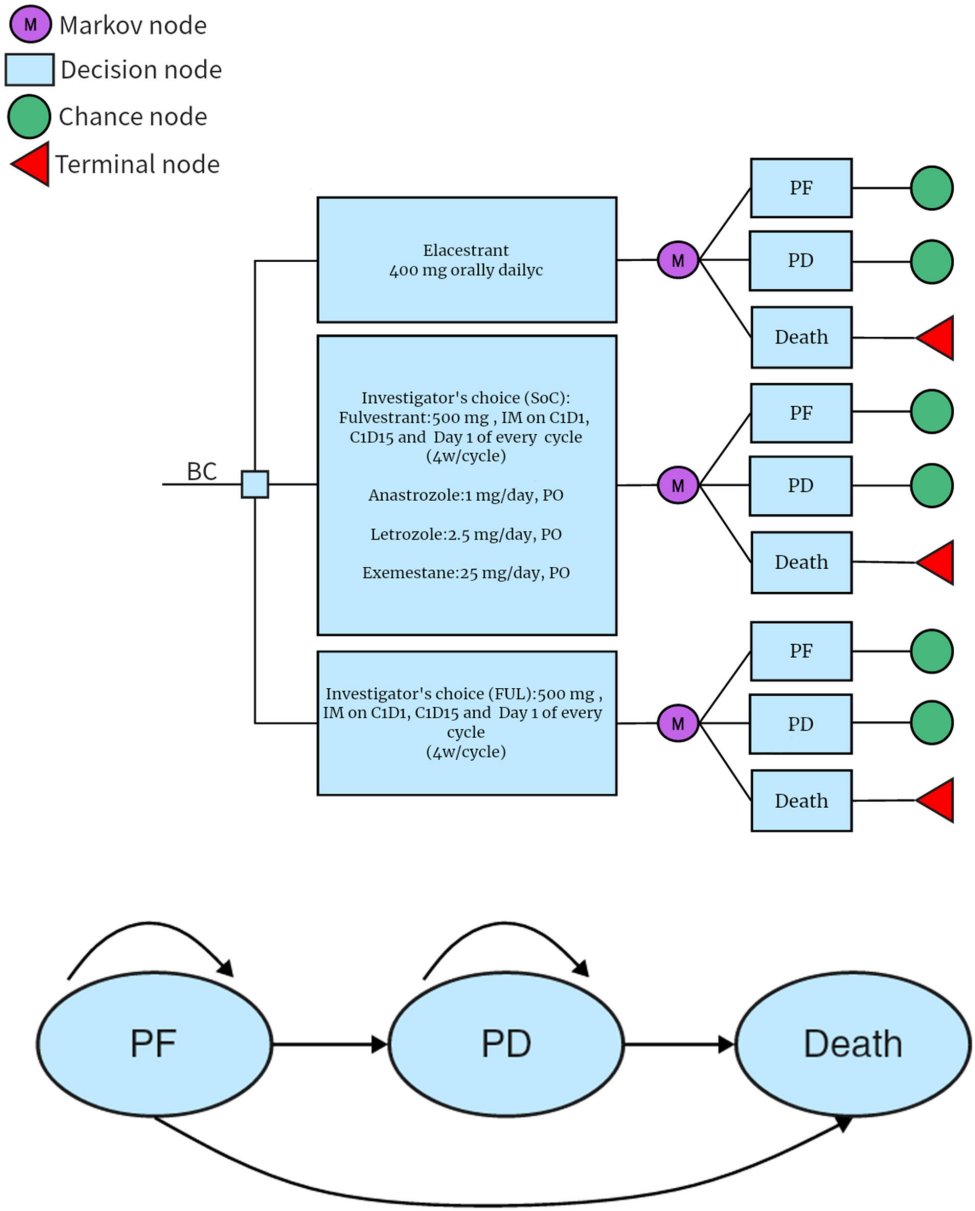
PSM simulating the results of the EMERALD clinical trial. All patients started in the PFD and received appropriate treatment. Patients could enter the PFD state and subsequently move to the death state. PD, progressed disease: PF, progression-free disease.

The PS model assumed that all patients were in the PF health state at the beginning and were able to maintain their particular health state or progress into healthy state in each cycle (**Figure 1**). The probability of the PF state transitioning to the death state was assumed to be natural mortality (35). The model was built by TreeAge Pro2022 software and analyzed statistically. The proportion of members was determined in each status from the survival curves base on the PS model. The cycle of the model was set to 1 month for the case of calculation, which was also consistent with the dosing schedule of FUL in EMERALD. The 5-year relative survival rate for women with metastatic breast cancer in the U.S. is 30%. The 5-year survival rate for men with metastatic breast cancer is 19%; thus, the time horizon was set to 10 years, which was sufficient to model an OS of patients with A/MBC (35). Patients entered the model and started cycling into different states until death, incurring treatment costs and health effects. The primary outcomes included total cost, quality-adjusted life-years (QALYs), and incremental cost-effectiveness ratio (ICER), which is expressed as the cost per QALY. All of them were discounted by 3% according to Weinstein M C et al.’s recommendations (36).

### 2.4 Cost

All relevant data have been listed in **Table 1**. As we adopted the perspective of American payers, we only consider direct healthcare costs, including drug acquisition costs, administration and medical fees for each state, end-of-life care costs, and costs associated with MAEs. The drug unit costs were obtained from the Centers for Medicare & Medicaid Services and AWP&AAC Medicaid, while all other costs were derived from published economic articles on similar drugs, and the cost per cycle was calculated. Since there is no clear median time for drug administration in EMERALD, it was assumed that the duration of treatments continues until the patients’ PD. According to the recommendations of the NCCN guidelines, progression after second/third-line treatment should be managed as supportive care. FUL monotherapy is administered via injection, thus drug management costs should also be taken into consideration. It was assumed equal opportunities for anastrozole, letrozole, or exemestane monotherapy by the investigator’s choice. Patients in the PF state require being followed up and monitored until disease progression, which mainly included laboratory scans and tests as well as bone metastasis treatment. The costs for patients in the PD include subsequent drug treatment costs and best supportive care costs, calculated by multiplying the cost of each cycle by the number of cycles. The impact of grade 3 or 4 adverse events (≥5%) and a difference in the incidence of >50% between the arms were considered in our study. The associated costs are sourced from published literature. The application of AE cost was limited to the first cycle of the model and assumes a monthly occurrence rate of only once.

### 2.5 Utility

The utility values for PF were derived from the study conducted by Mistry et al. (32). The utility was calculated using the latest UK value set, with data collected from the EuroQol 5-Dimension 5-Level (EQ-5D5L) data collected in the MONALEESA-2 trial. The utility values for PD were sourced from the study reported by Lloyd et al. (33), which used standard gambling techniques to report estimated health state utility values. The disutilities of adverse events were obtained from published literature. The calculation of the MAEs per cycle’s disutilities was determined by multiplying the probability of the AE with its corresponding utility.

### 2.6 Sensitivity analyses

The impact of different parameters on the stability of the results was evaluated using one-way sensitivity analysis. The prices and variations of ELA, FUL, Anastrozole, Exemestane, and Letrozole were determined based on the FDA recommendations and existing market prices. Administration cost, follow-up cost, adverse event cost, utilities, and discount rates were obtained from published literature. The variation range of the remaining parameters was set at 20%. The results were presented in the form of tornado diagrams. As the drugs used in EMERALD were of fixed dosage, changes in body surface area and weight were not considered.

A second-order Monte Carlo simulation was used for probabilistic sensitivity analysis. Based on the recommendation of the ISPOR-SMDM Modeling Good Research Practice Working Group, costs, incidence of MAEs as well as all utilities were set to gamma, beta, and normal distributions, respectively (37). The utility and the transition probability parameter were assumed to conform to the β distribution, and the cost parameter was assumed to conform to the γ distribution (Briggs et al, 2012) (37). Probabilistic sensitivity analysis (PSA) was conducted with 1000 iterations to examine parameter uncertainty in the entire model. The results were presented in the form of cost-effectiveness acceptability curves and an incremental cost-effectiveness scatter plot. According to the suggestion of Neumann et al., the willingness-to-pay (WTP) threshold for the United States is $150,000 (38).

## 3 Results

### 3.1 Base case results

Our study only compared the cost-effectiveness analysis of ELA with SOC. In terms of incremental costs and QALYs, in the overall patient group, ELA increased by0.08 QALY compared to SOC. Additionally, the incremental cost of ELA was $754,158, resulting in an ICER increase of $8,672,360/QALY for the overall population. While in the subgroup, ELA increased by 0.51 QALY compared to SOC. It was associated with the additional cost of $906,533, which led to an ICER of $2,900,560/QALY. (Shown in **Table 2**) Both ICER values were significantly higher than the threshold value of $150,000/QALY. As for the life years, ELA had an additional 0.01 compared to SOC. while in the subgroups, ELA had an additional 0.78 compared to SOC. which were consistent with the results observed in EMERALD, validating the model.

**Table 2:** The results of the base case analysis.

	All patients			Patients with ESR1 MUTATION		
	ELA	SOC	FUL	ELA	SOC	FUL
Total cost ($)	1,260,727	506,569	505,473	1,421,188	416,064	514,654
Incremental costs ($)	754,158	1,096	–	906,533	–	98,590
Total effectiveness (QALYs)	1.36	1.28	1.27	1.59	1.08	1.27
Incremental effectiveness (QALYs)	0.09	0.00	–	0.31	–	0.19
ICER ($/QALY)	8,672,360	236,938	–	2,900,560	–	509,831
LYs	2.53	2.52	2.51	2.88	2.10	2.53

QALYs, quality-adjusted life years; SOC, standard-of-care; Lys, life years; ELA, elacestrant; FUL, fulvestrant.

### 3.2 One-way sensitivity analysis

**Figure 2:**
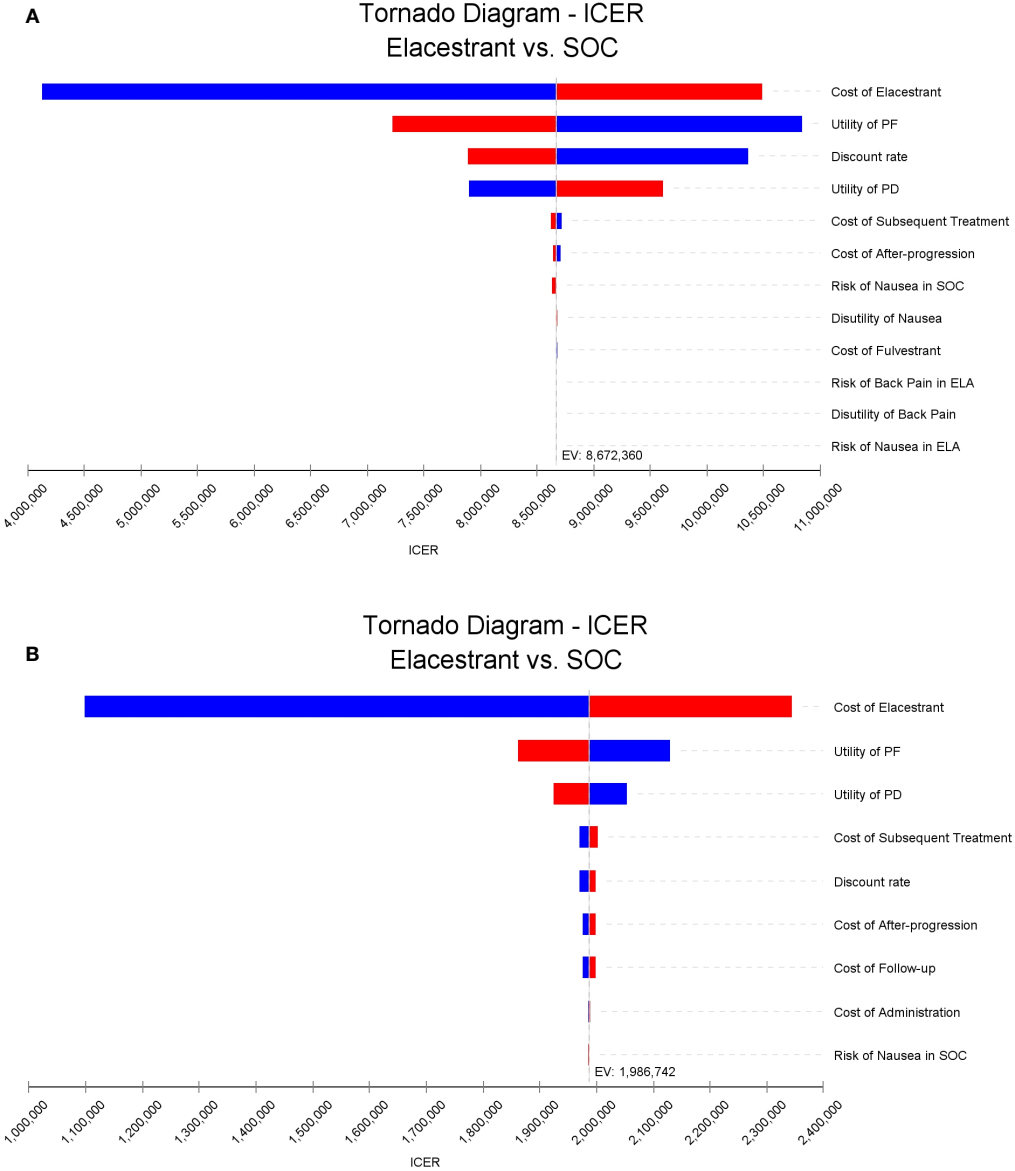
Tornado diagrams of one-way sensitivity analyses. **(A, B)** were the results for the overall population and the subgroup, respectively. The dotted line intersecting the blue and red bars represents the ICER of base case results. ICER, incremental cost-effectiveness ratio.

The results of the one-way sensitivity analysis were shown in **Figure 2**. The key model drivers was the cost of ELA, followed by the utility values of PF and PD in both the overall group and subgroup. Other costs such as subsequent treatment cost, cost of after-progression, follow-up and administration, and some additional parameters including the discount rate, and risk of MAEs, such as nausea in SOC also had a slight impact on the ICER.

### 3.3 Probabilistic sensitivity analysis

**Figure 3:**
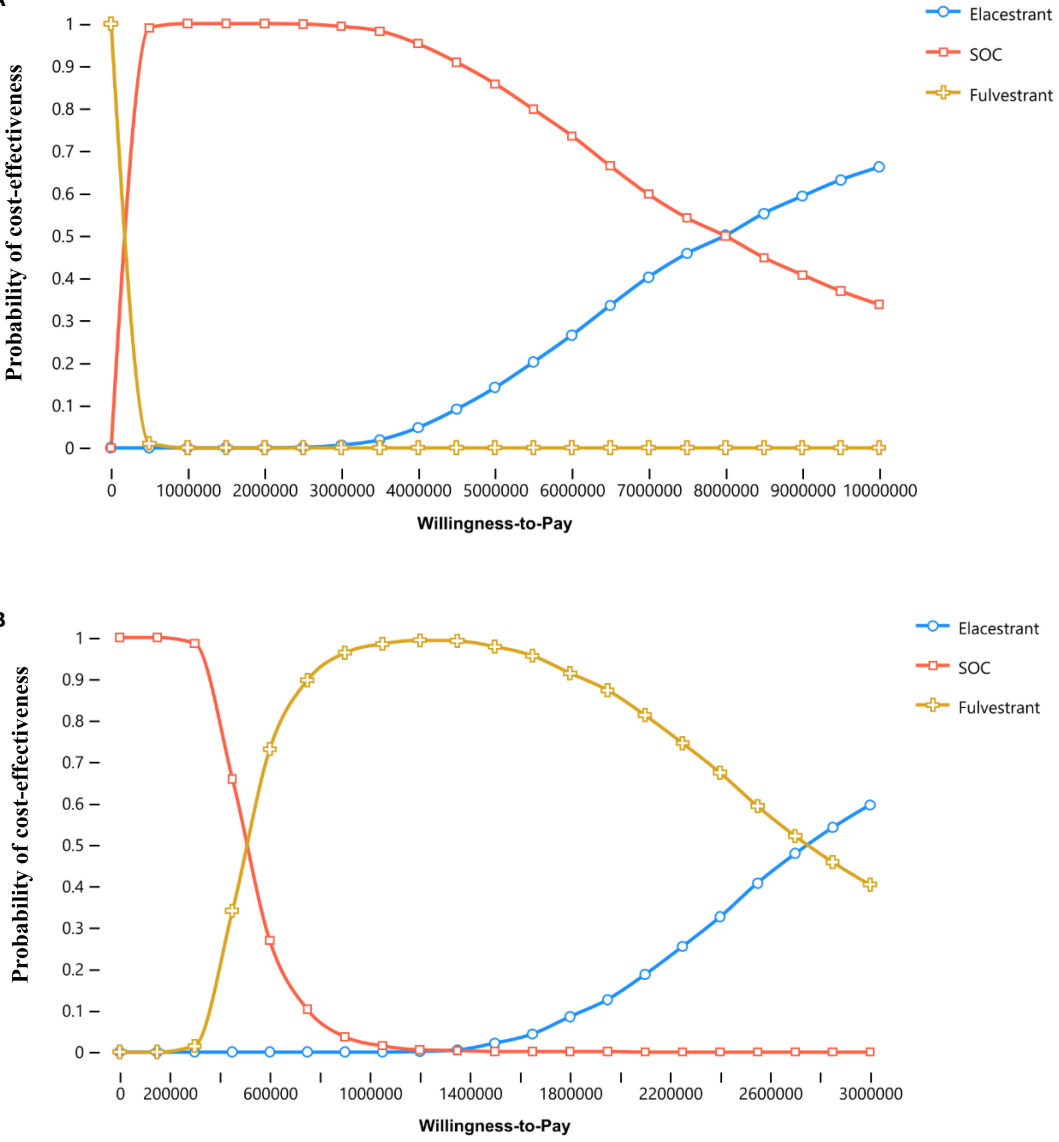
Cost-effectiveness acceptability curves. **(A, B)** were the results for the overall population and the subgroup, respectively.

In both overall population and subgroup, the probability of ELA being cost-effective *vs*. SOC or FUL at thresholds of $150,000 per QALY gained was 0%. The cost-effectiveness acceptability curves for three treatments were shown in **Figure 3**.

## 4 Discussion

Endocrine therapy stands out as a highly effective treatment for ER+ breast cancer. Nevertheless, the persistent challenge of endocrine resistance in advanced ER+ breast cancer complicates the clinical landscape. Early approved endocrine therapies fall into broad categories, including AIs, selective estrogen receptor modulators (SERMs), and SERDs. These therapies can be utilized with or without ovarian suppression. AIs, administered orally, play a pivotal role in reducing the risk of relapse post curative therapy and represent the standard first-line treatment for metastatic disease. Often, they are employed in conjunction with a CDK4/6 inhibitor. However, AIs are not without side effects, including the exacerbation of menopausal symptoms, vaginal dryness, arthralgia, and accelerated bone loss. Clinical challenges associated with SERDs, like FUL, involve the dual action of antagonizing endoplasmic reticulum transcriptional activity and promoting its degradation. Nevertheless, pharmacologic limitations, including a lack of oral bioavailability, intramuscular injection administration with low patient compliance, and arthralgia-related side effects, impede their widespread use. The activity of FUL or AIs in the context of ESR1 mutations remains incompletely characterized due to limited retrospective datasets. The combination of the low oral bioavailability of FUL and the necessity for intramuscular administration underscores the demand for a more effective oral SERD (17, 39).

The EMERALD study provided a new treatment option for patients with advanced/metastatic ER-positive/HER2-negative breast cancer who had experienced progression after previous endocrine therapy and CDK4/6 inhibitor treatment. It demonstrated that ELA was the first oral SERD that significantly improved PFS compared to SOC. In this study, we evaluated the cost-effectiveness of ELA versus standard-of-care. According to current endocrine therapy guidelines (10), priority was given to AIs and FUL, and there have been related articles on the economy showing that FUL monotherapy was the most cost-effective (in the absence of a combination of drugs) (20). Due to the representation of FUL, we chose to take FUL out from the SOC group and compare it with ELA separately, which was also consistent with the trial design of EMERALD.

Compared with intramuscular injection of FUL, ELA has better therapeutic effects and a more universal and patient-compliant oral administration method. Nevertheless, ELA is a relatively expensive drug. The basic results of this study showed that compared with SOC and FUL, the ICER values of ELA in the overall population and subgroup were $8,672,360/QALY and $2,900,560/QALY, respectively, both of which were significantly higher than the WTP threshold of $150,000/QALY. Accordingly, under the WTP threshold of $150,000/QALY, ELA did not have an economic advantage, indicating that ELA was not a cost-effective choice under the payment willingness of Americans.

In the United States, the traditionally accepted threshold for the cost-effectiveness ratio is $50,000/QALY (38). In basic case analysis, ELA has been found to increase QALYs by 0.09 compared to SOC in the overall population. This was due to ELA extended PFS to a certain degree, and the risk ratio of OS in EMERALD was 0.75, indicating that ELA had an effect in reducing the risk of disease progression or death. In the subgroup analysis, ELA showed a superior effect compared to FUL, with an increase in QALYs of 0.31 and a LYs of 2.88. This was due to ELA’s advantage in filling the therapeutic gap for patients with ESR1 mutations. Clinical trial results also indicated that the improvement in PFS may be lower in patients without ESR1 mutations (11). ELA had higher ICER values in both groups, the potential reason was somewhat associated with a higher incidence of MAEs compared to traditional drugs (treatment-related grade 3/4 MAEs occurred in 7.2% receiving ELA and 3.1% receiving SOC). This article did not delve into the economic comparison between SOC and FUL.

The sensitivity analysis of the two groups indicated that, the model was more sensitive to the cost of ELA. Apart from the utility of PF and PD, the cost of subsequent treatment and cost after progression had the greatest impact on the model results. It may result from the fact that the patients included in the clinical trial had already received first/second-line treatment and deteriorated. All patients who entered the PD state in the clinical trial received subsequent treatment until death, or intolerant patients were directly referred to the next level of treatment. Thus, the selection of drugs, the requirements of the medical environment, and the consumption of medical supplies were all more sophisticated and professional, resulting in higher costs.

To the best of our knowledge, this is the first study to explore the cost-effectiveness of ELA This paper evaluated, for the first time, the economic viability of ELA as a treatment for ER-positive/HER2-negative advanced or metastatic breast cancer patients using economic modeling methods. The findings offered the latest evidence for the formulation of relevant medical insurance policies and clinical decisions. However, our research also has some limitations. Firstly, in terms of the collection of cost-effectiveness data on MAEs, no matching reports on nausea were found in the articles on the second-line treatment of breast cancer. Therefore, we used articles on advanced esophageal squamous cell carcinoma and advanced non-small cell lung cancer as the utility and cost parameters of the model, respectively, while the utility of back pain was selected from a Canadian study. Secondly, the management costs of grade 1 and 2 adverse events were not included in this study. However, the result of sensitivity analysis showed that these parameters only have a slight impact. Secondly, EMERALD did not provide the median dosing time, which has caused certain deviations in our drug-cost calculation results. Since the median dosing time was unknown, it was assumed that all patients would receive the assigned drug until PD. Such calculations may not correspond with the actual clinical process. Furthermore, in the SOC group, EMERALD did not provide the proportion or number of per investigator’s choices for all drugs except for the number of FUL users. Therefore, for convenience in the calculation, we assumed that the number of patients receiving anastrozole, letrozole, or exemestane monotherapy was the same. These patients were divided into three groups and given the drugs above separately. Nevertheless, such an assumption may deviate from the actual clinical design, and cost calculations may lead to certain biases. Thirdly, after consulting with physicians, we learned that the question of how to proceed with subsequent treatment after disease progression following ELA therapy was extremely complex because it depended on the first/second-line therapeutic regimen. Notably, NCCN has not yet provided new recommendations for subsequent treatment for patients who have progressed after ELA therapy so far. Accordingly, regarding costs other than drug and adverse reaction expenses, we referred to data from pharmacoeconomic articles on FUL (20), which was also a second-line treatment for A/MBC. Whereas, another issue arose: published economic evaluations of the same type of drugs so far required that the patients had not received CDK4/6 inhibitor treatment, which partly deviates from the inclusion criteria of our study. Consequently, the cost of subsequent treatment we reference may include the cost of CDK4/6 inhibitors (40). In the end, though PSM is one of the most popular methods in oncology evaluation including the evaluation of drugs for leukemia treatment currently, the limitations of the PSM arise from its assumption that the survival function is independent. Although the conceptual model includes transitions between different health states, the implemented structure does not explicitly model the disease or estimate transition probabilities for all possible transitions. Therefore, it is incorrect to describe the PSM as a state transition model, as it does not establish a structural connection between health states or estimate transition probabilities for each possible transition. Also, the sensitivity analysis cannot account for variations in drug effectiveness unless bootstrapping is employed (41, 42).

The purpose of this study is to compare the new endocrine therapy with the existing endocrine therapy, rather than evaluating combination therapy. The benefits of ELA relative to FUL and AIs monotherapy in the EMERALD trial also suggest that ELA was a promising strategy as a preferred endocrine backbone therapy in future early combination studies. Therefore, further clinical studies are necessary to evaluate the economic feasibility of comparing ELA/everolimus with exemestane/everolimus combination and ELA/alpelisib with FUL/alpelisib combination. Finally, our team is looking forward to the ultimate OS results being provided when the data is mature in the future so that researchers can obtain more complete data to conduct economic evaluations more professionally and accurately.

## 5 Conclusion

Based on cost-effectiveness analysis and sensitivity analyses, the results indicate that under the WTP threshold of $150,000, ELA is not a cost-effective option compared to the standard-of-care for second-line treatment of advanced or metastatic ER-positive/HER2-negative breast cancer patients in the United States.

## Data availability statement

The original contributions presented in the study are included in the article/**Supplementary Material**. Further inquiries can be directed to the corresponding authors.

## Author contributions

WZ: Formal Analysis, Investigation, Software, Writing – original draft. XC: Conceptualization, Resources, Writing – review & editing. JL: Data curation, Methodology, Writing – original draft. BZ:Validation, Writing – review & editing. NL: Validation, Writing – review & editing. ML: Visualization, Writing – review & editing. HC: Conceptualization, Project administration, Supervision, Writing – review & editing.
