# Supplementary material for: Cost-effectiveness analysis of elacestrant versus standard endocrine therapy for second-/third-line treatment of patients with HR+/HER2- advanced or metastatic breast cancer: a US payer perspective
Source: Front Oncol. 2023 Dec 19;13:1272586. doi: 10.3389/fonc.2023.1272586 (PMC10758478; doi:10.3389/fonc.2023.1272586)
Supplement: Supplementary file 1 [file DataSheet_1.docx]

**Appendix A** (see the abbreviations at the end of the document)

**Supplementary Figure S1.** The replicated Kaplan-Meier survival curves for the OS curves in overall population and subgroup. Figures A&B were results of elacestrant and SOC in overall arms. Figures C&D were the results of elacestrant and SOC in ESR1 mutation arms.


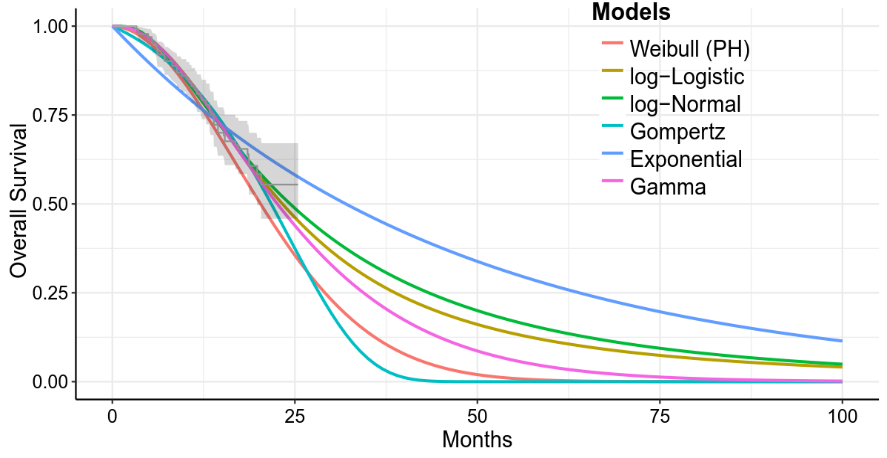

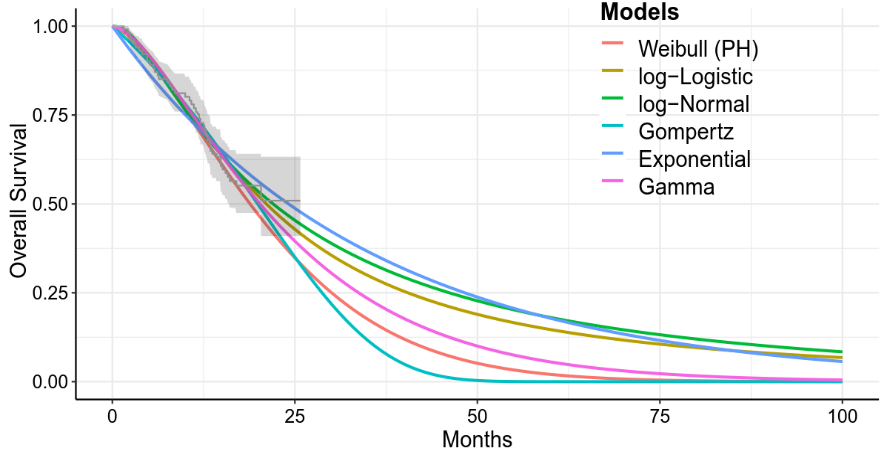

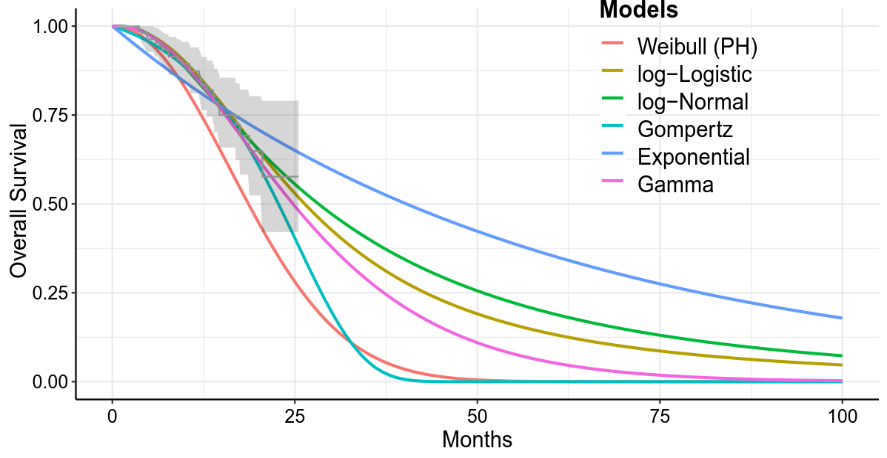

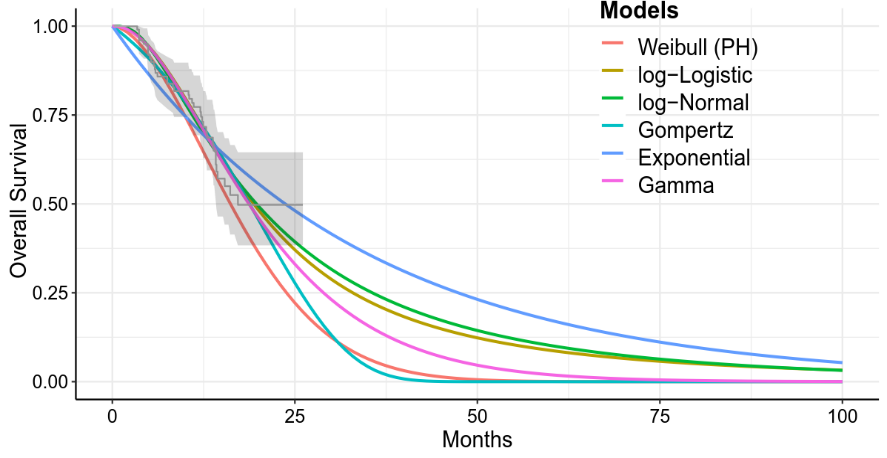


A

B

C

D

A

B

C

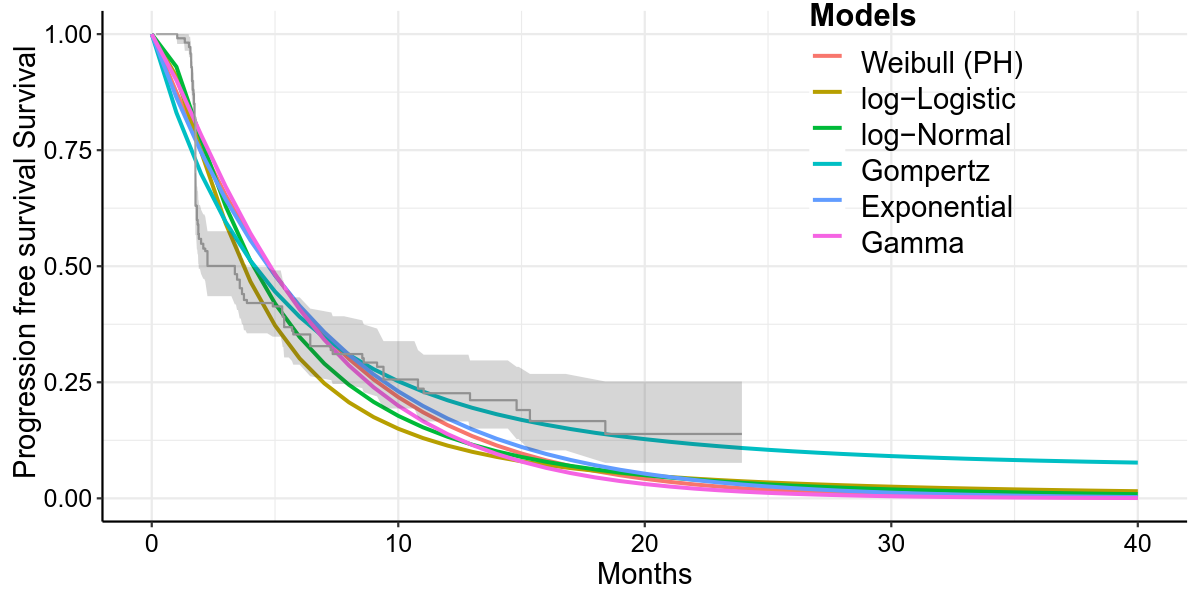

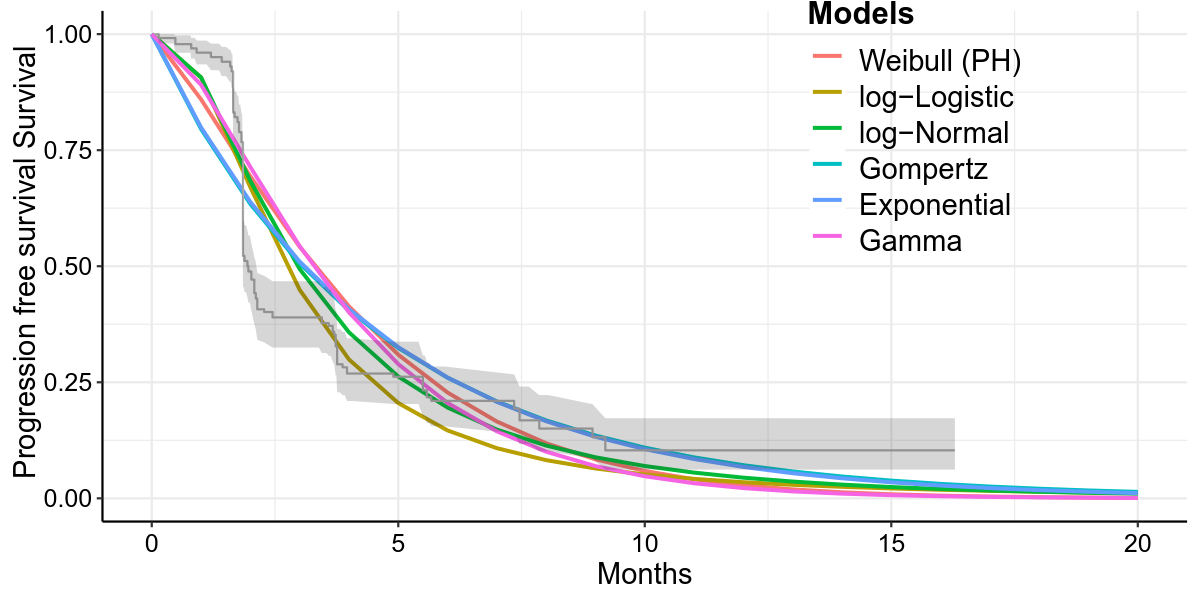

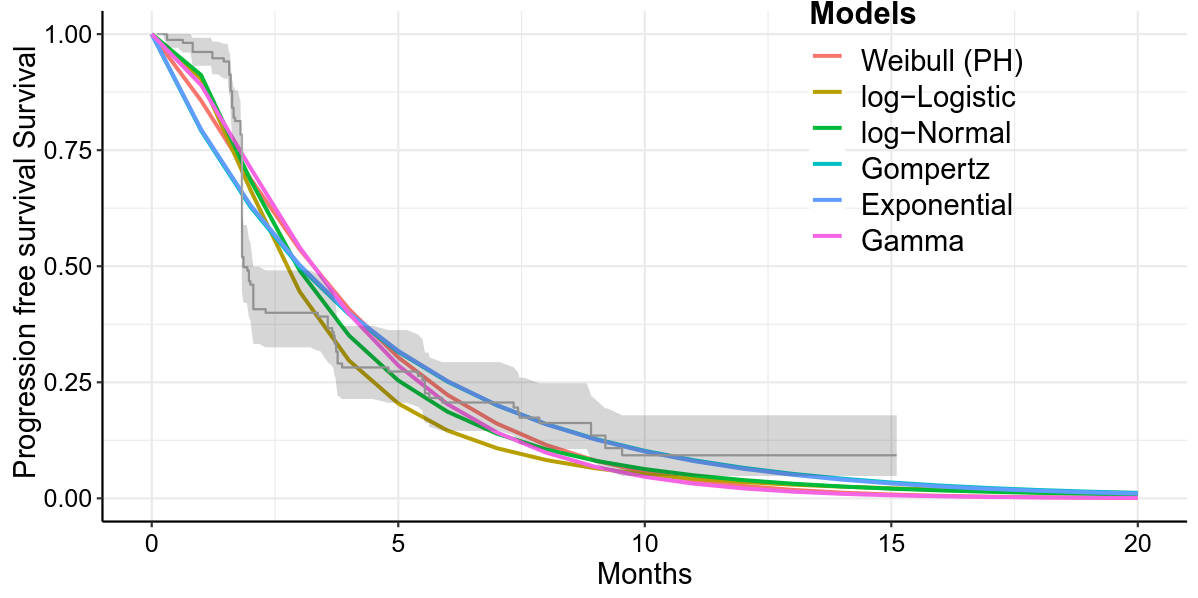

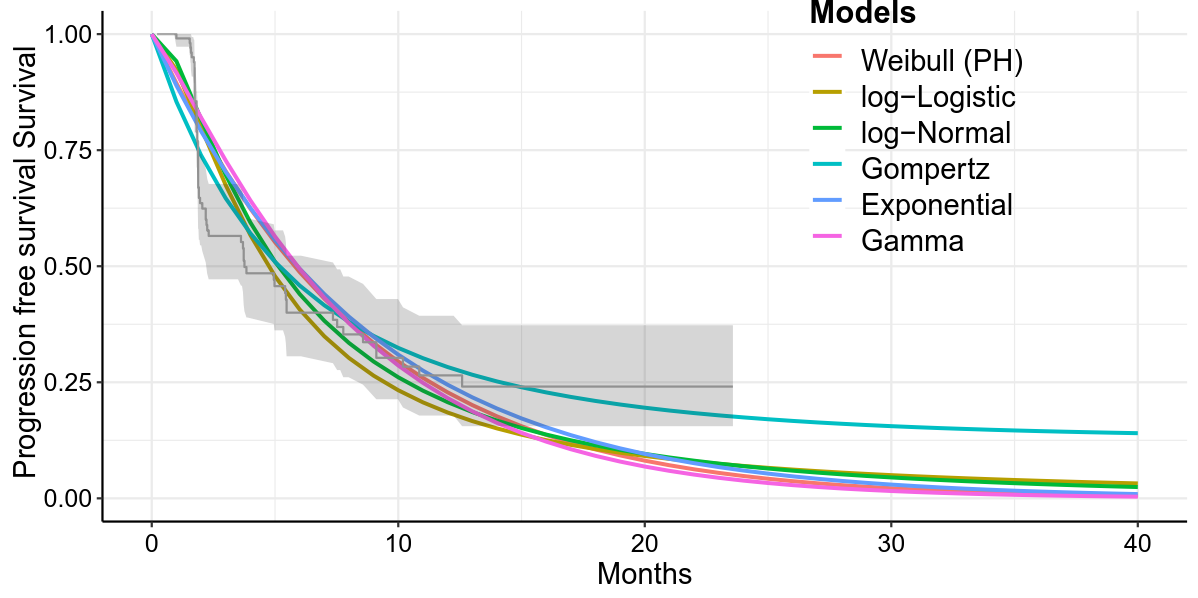


D

**Supplementary Figure S2.** The replicated Kaplan-Meier survival curves for the PFS curves in overall population and subgroup. Figures A-C were the results of elacestrant, SOC and fulvestrant in overall population, respectively. Figures D-F were the results of s elacestrant, SOC and fulvestrant in ESR1 mutation arm, respectively.

E

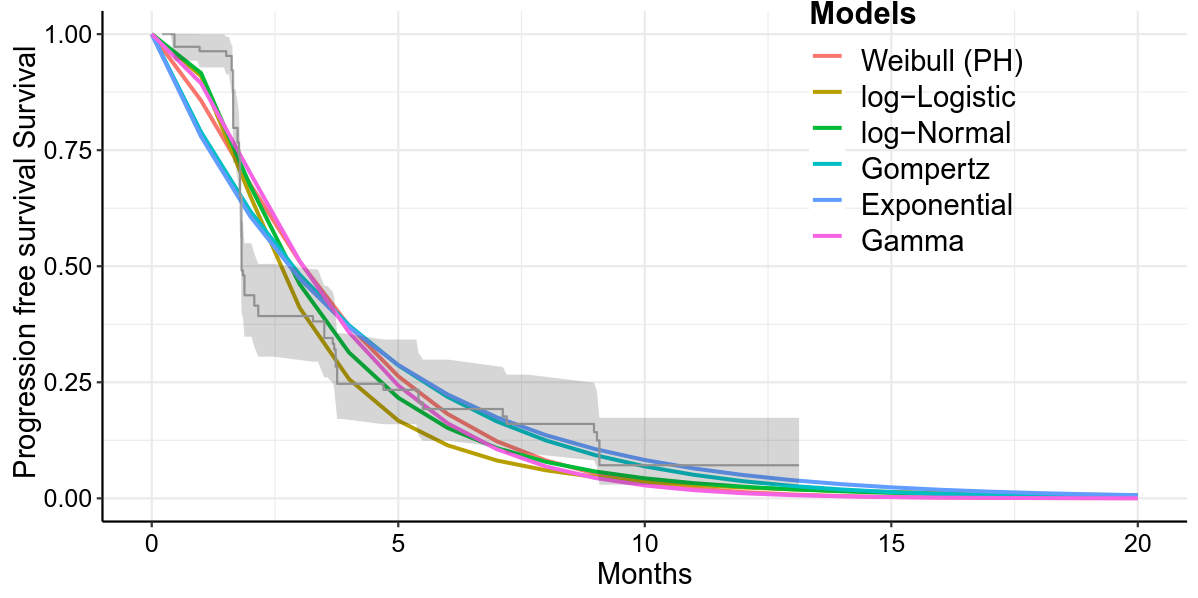

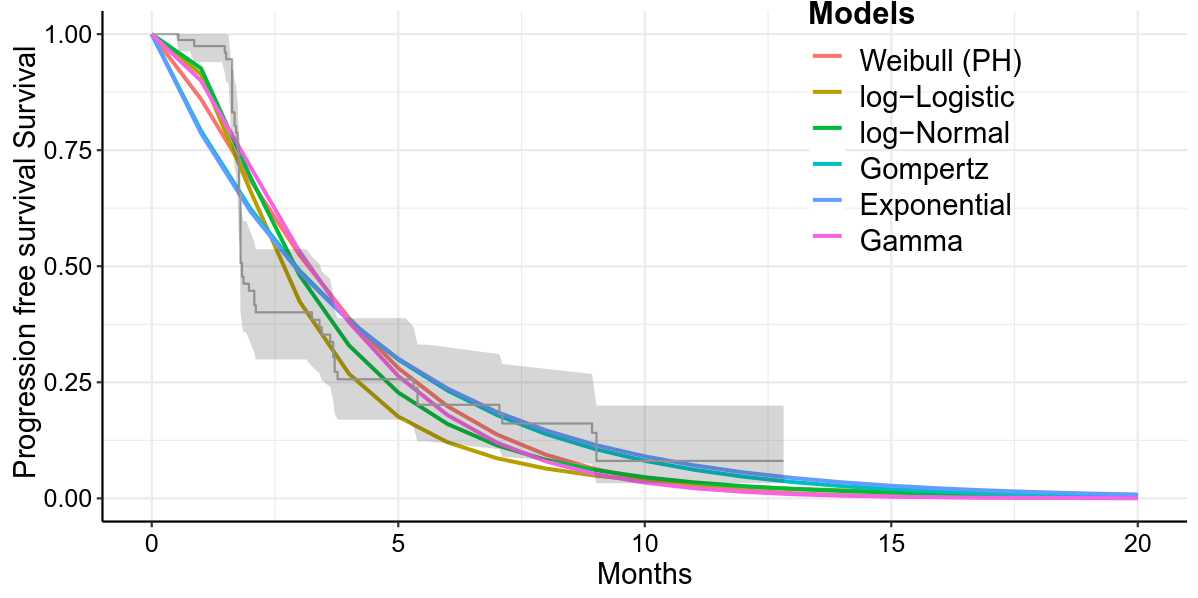


F

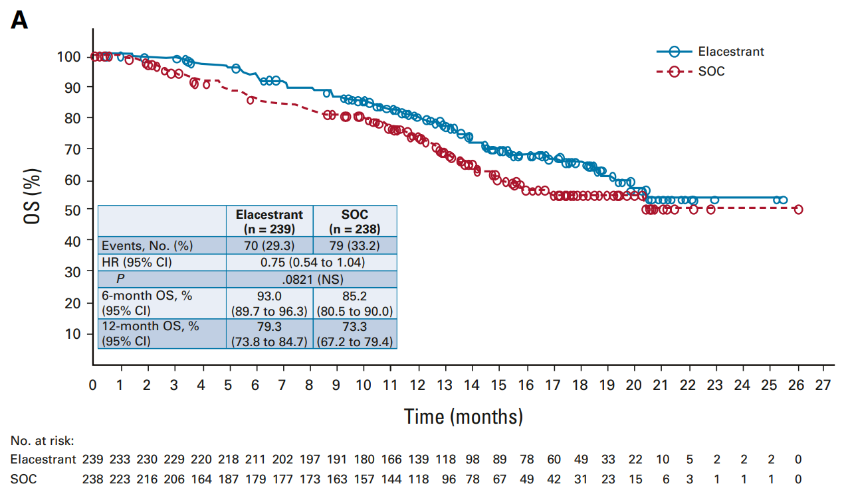

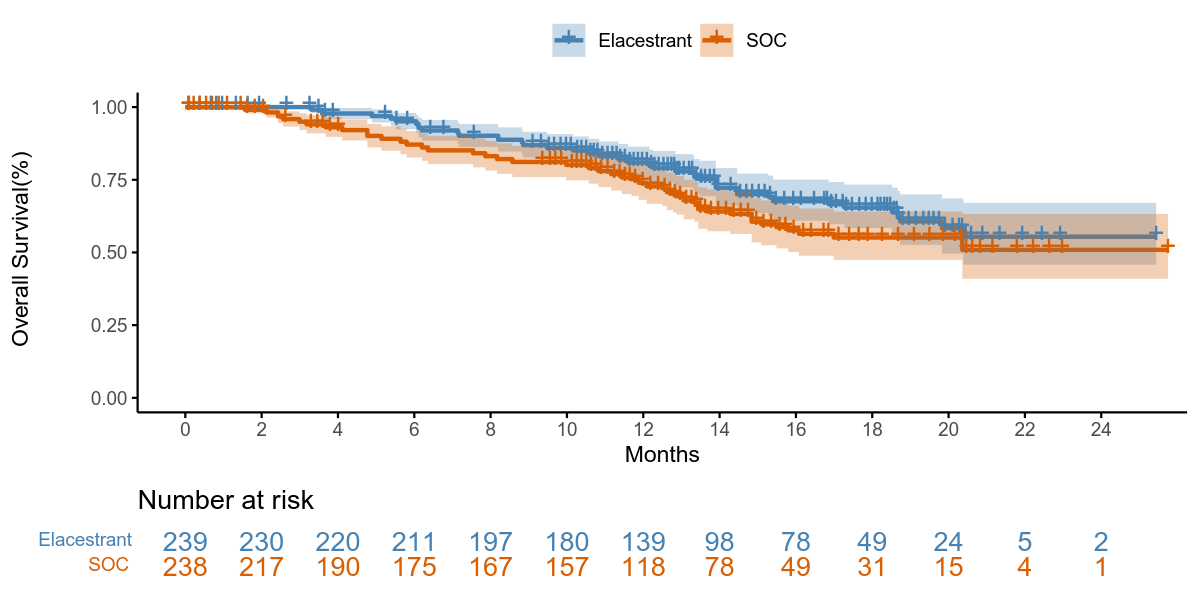


**
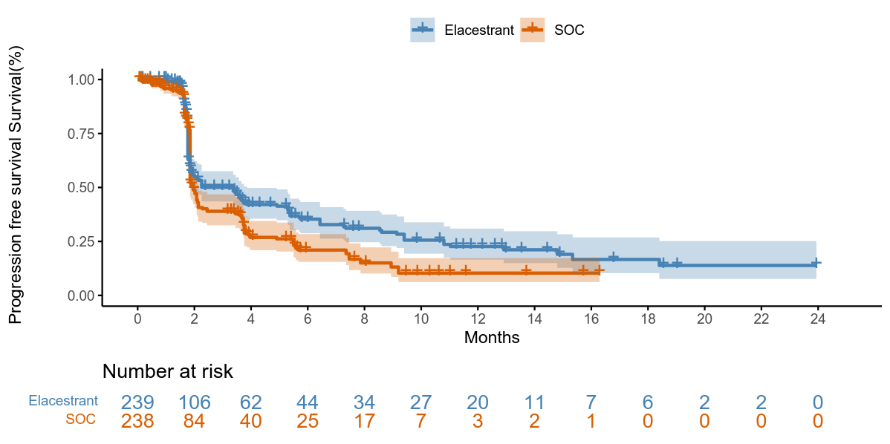
**


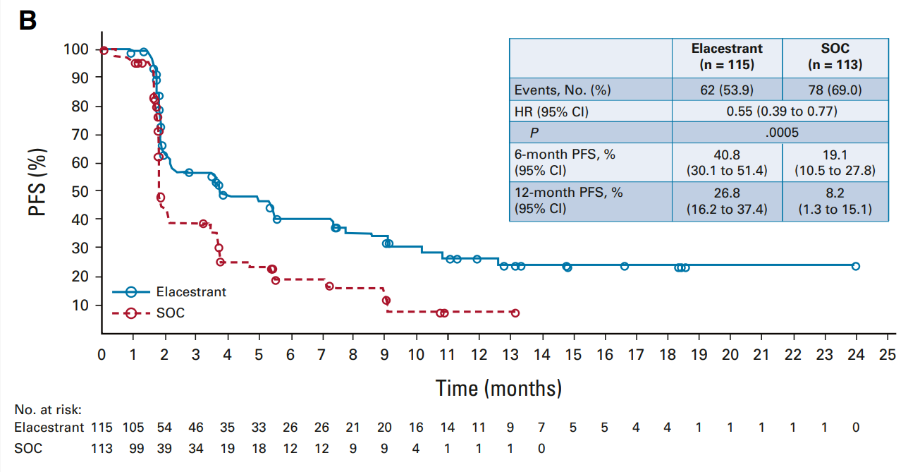

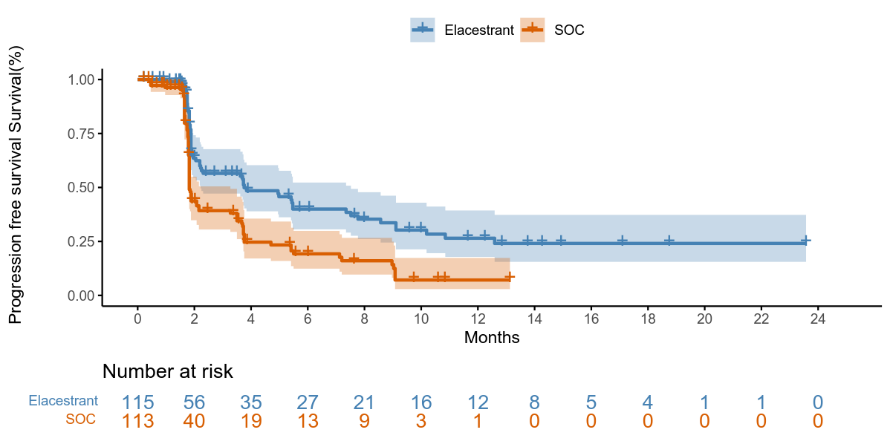


D


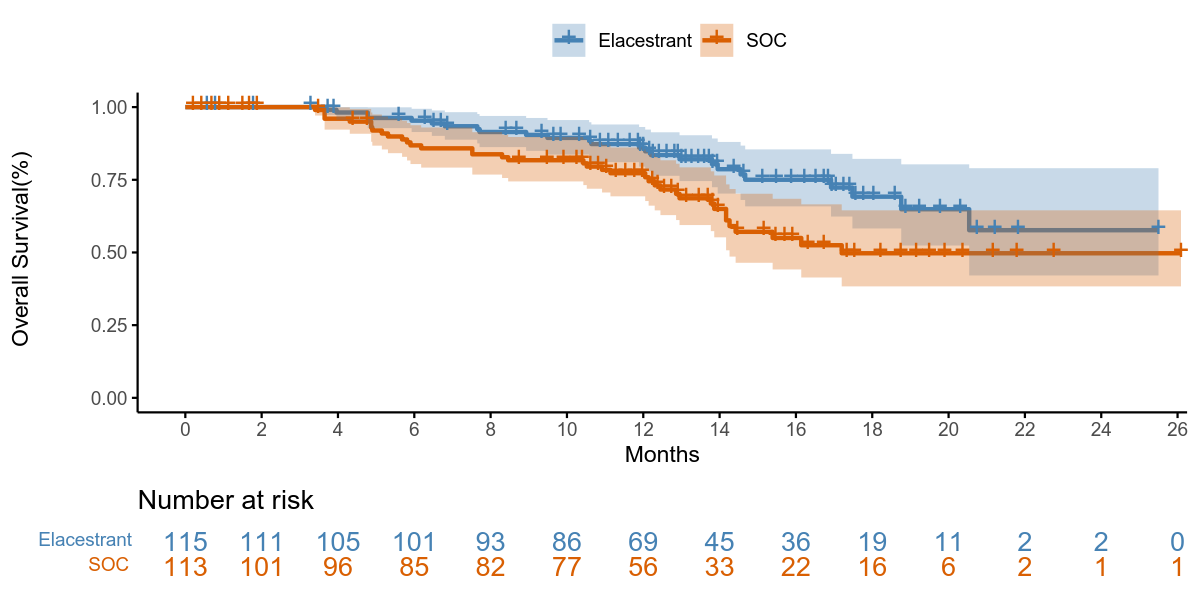

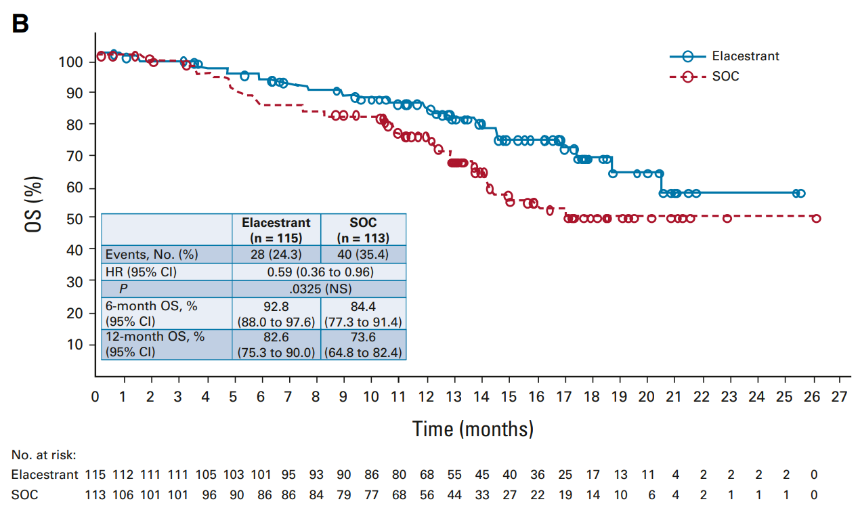

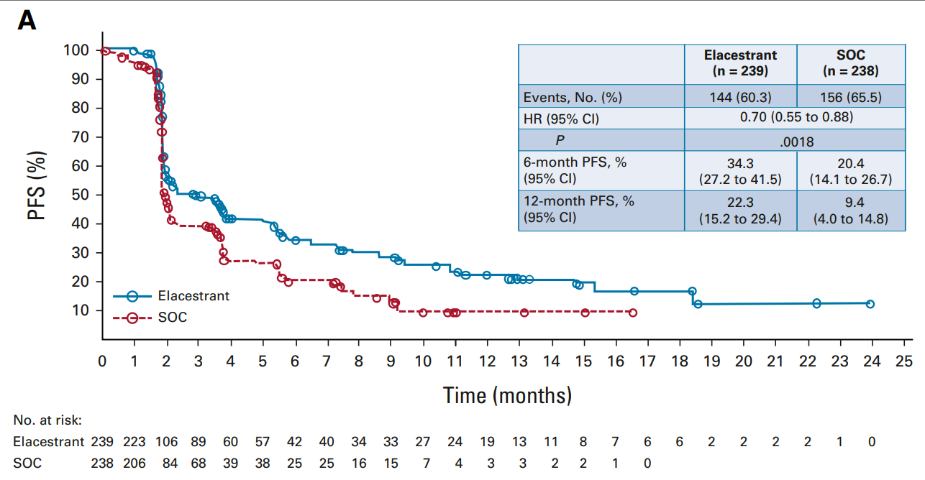


C


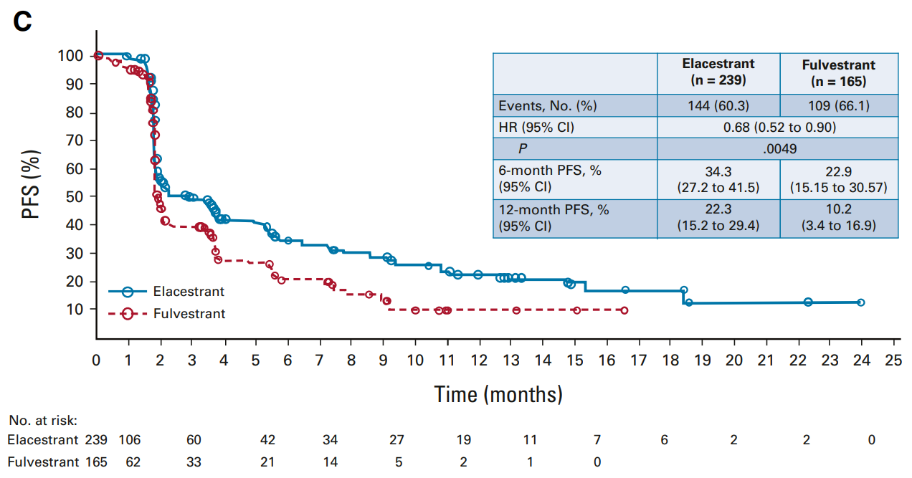


E


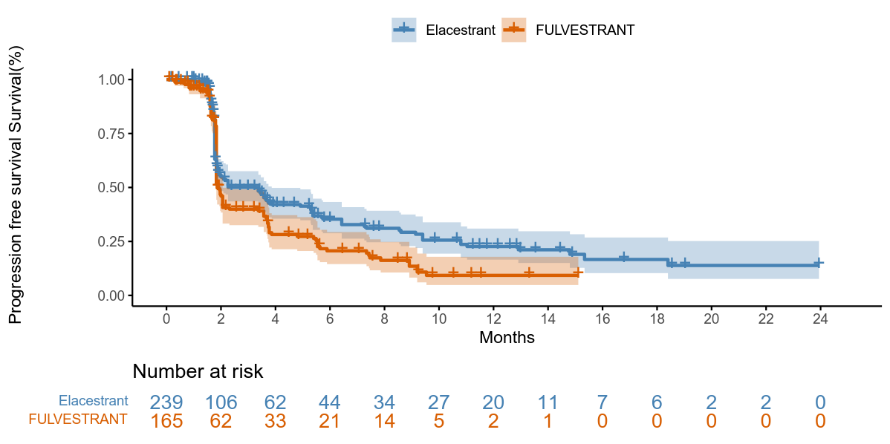

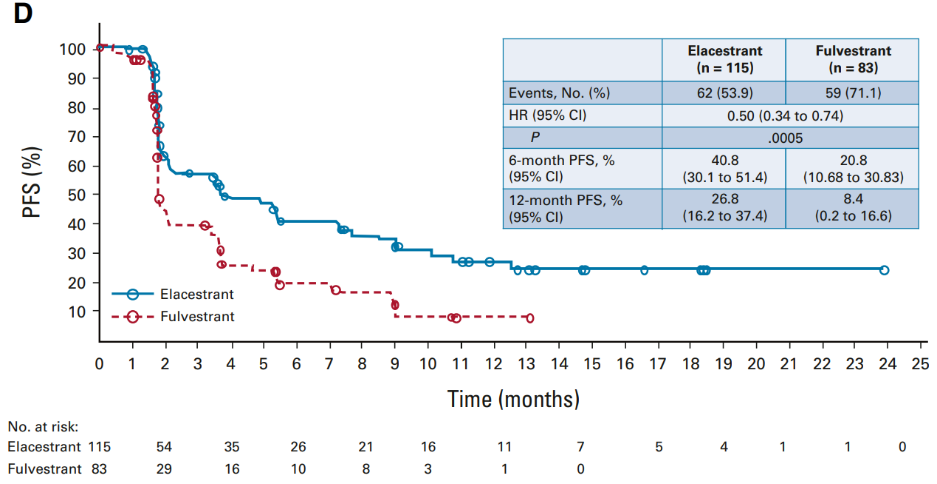


F


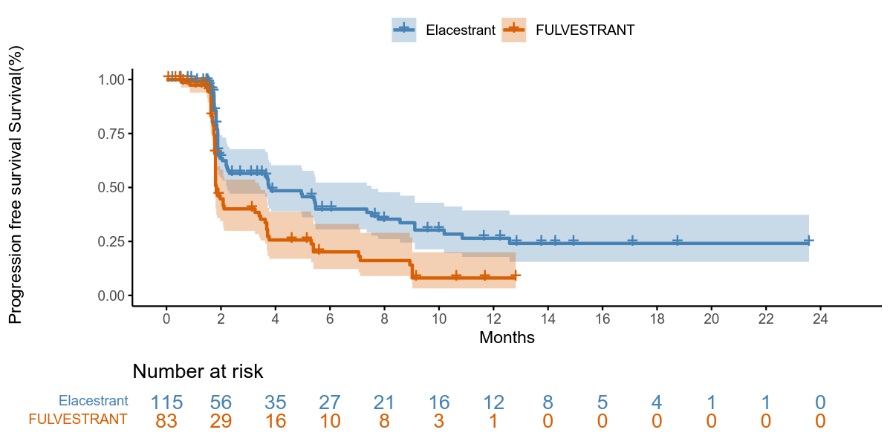


**Supplementary Figure S3.** Kaplan-Meier survival curves of arms elacestrant, SOC and fulvestrant in the EMERALD clinical trial compared to corresponding fitting models.

**Supplementary Figure S4.** Incremental Cost-effectiveness Scatter Plot of Monte Carlo simulations for 1,000 steps. Figures A&B were the results of the overall population arms. Figures C&D were the results of ESR1 mutation arms.


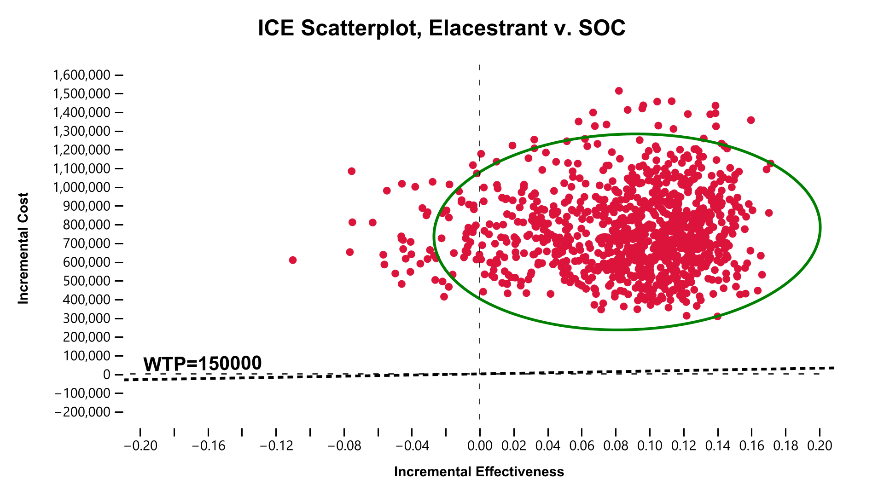

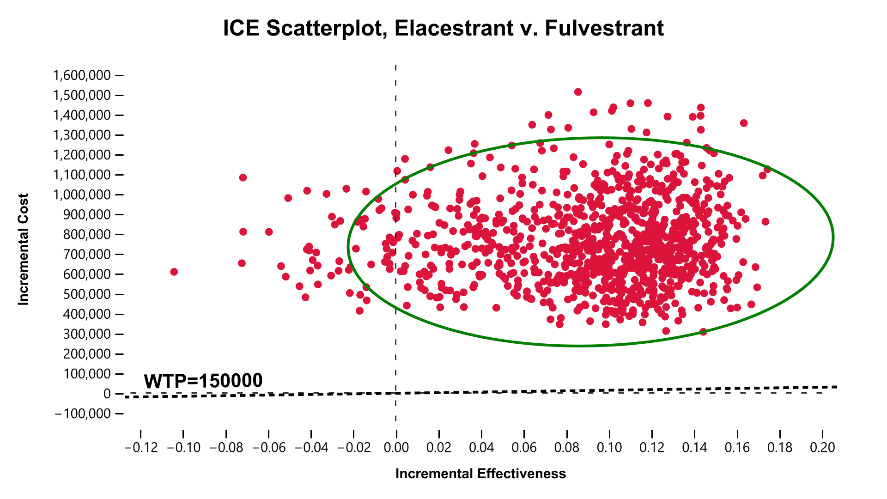

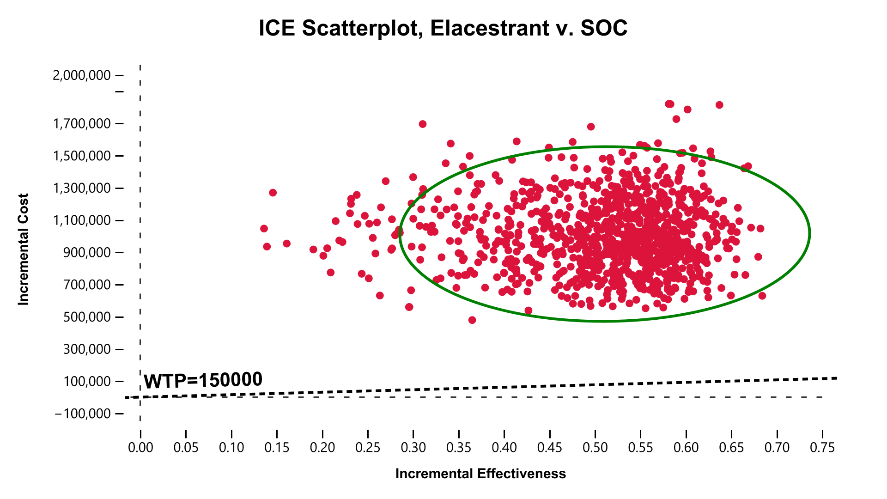

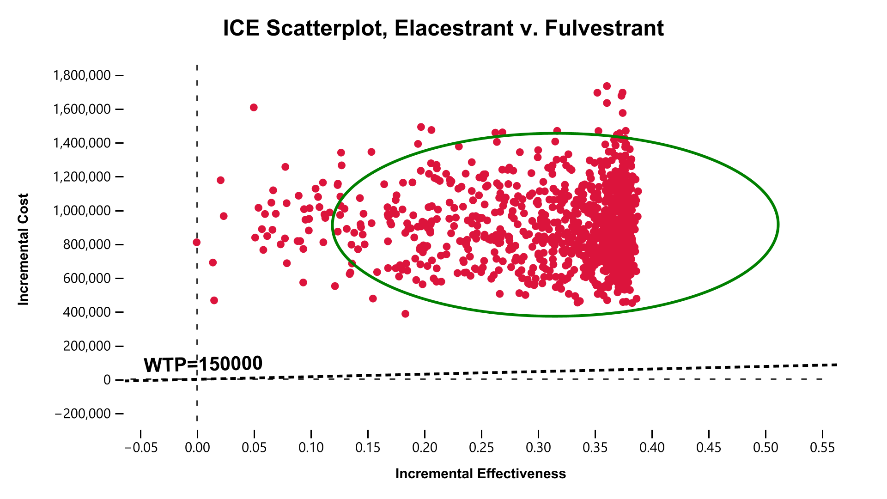


D

C

A

B

The following abbreviations are used in the supplementary material:

**SOC:** standard of care

**OS:** overall survival

**PFS:** progression-free survival

**WTP:** willingness-to-pay
